# Longitudinal individual predictions from irregular repeated measurements data

**DOI:** 10.1038/s41598-022-26933-1

**Published:** 2023-01-18

**Authors:** Iris Eekhout, Stef van Buuren, Bram Visser, Marco C. A. M. Bink, Abe Huisman

**Affiliations:** 1 The Netherlands Organization for Applied Scientific Research (TNO), Child Health, Leiden, The Netherlands; 2 Department of Methodology and Statistics, Utrecht University, Utrecht, The Netherlands; 3 Hendrix Genetics, Boxmeer, The Netherlands

**Keywords:** Epidemiology, Computational models, Data processing, Predictive medicine, Software, Statistical methods, Computational biology and bioinformatics

## Abstract

Intensive longitudinal data can be used to explore important associations and patterns between various types of inputs and outcomes. Nonlinear relations and irregular measurement occasions can pose problems to develop an accurate model for these kinds of data. This paper focuses on the development, fitting and evaluation of a prediction model with irregular intensive longitudinal data. A three-step process for developing a prediction tool for (daily) monitoring and prediction is outlined and illustrated for intensive weight measurements in piglets. Step 1 addresses a nonlinear relation in the data by developing and applying a normalizing transformation. Step 2 addresses the intermittent nature of the time points by aligning the measurement times to a common time grid with a broken-stick model. Step 3 addresses the prediction problem by selecting and evaluating inputs and covariates in the model to obtain accurate predictions. The final model predicts future outcomes accurately, while allowing for nonlinearities between input and output and for different measurement histories of individuals. The methodology described can be used to develop a tool to deal with intensive irregular longitudinal data that uses the available information in an optimal way. The resulting tool demonstrated to perform well for piglet weight prediction and can be adapted to many different applications.

## Introduction

With current technology it becomes easy to register intensive longitudinal data, where each object of study is followed on many repeated time measurements. Such data can be used to explore important associations and patterns between various types of inputs and outcomes, to steer dynamic processes over time, or to enable individual prediction for the long-term based outcomes given a few measurements at start.

This paper focuses on the development, fitting and evaluation of a prediction model with irregular intensive longitudinal data. The development of prediction models poses several potential challenges. One challenge is to deal with possibly nonlinear associations between inputs and outcomes, which could complicate model development. Another challenge is that measurements for different individuals are taken at different time points^1^. For example, one individual may have measures at day 1, day 5 and day 20, whereas another individual may have measures at day 2, day 10 and day 25. Measurement times are not aligned here, and when we want to predict a later outcome based on repeated previous measurements, this needs to be resolved prior to making the prediction model. A third challenge is to define and select those predictors that will lead to accurate predictions.

In this paper we outline a three-step process for developing a prediction tool for (daily) monitoring and prediction. Step 1 addresses a nonlinear relation in the data by developing and applying a normalizing transformation. Step 2 addresses the intermittent nature of the time points by aligning the measurement times to a common time grid. Step 3 addresses the prediction problem by selecting and evaluating covariates in the model to obtain accurate predictions. The final model should be able to predict future outcomes as accurate as possible, while allowing for nonlinearities between input and output and for different measurement histories for individuals.

The development of the methodology is illustrated by an empirical data example of intense weight measurements in piglets. The piglets were weighted daily, and the interest is to predict piglets weight at the age of 160 days from earlier weight measures, e.g., from measurements during the first 14 days. The relations between age and piglet weight are nonlinear. Moreover, the measurements are taken at different days in different settings, and each piglet has an individual growth pattern that deviates from the mean pattern.

## Methods for modelling strategy

### Nonlinear relation between age and outcome

A common way of modeling nonlinear relations is to transform the data such that the relations between the transformed data is approximately linear^2^. For example, reference standards for height and weight in children are commonly used to monitor the well-being of children^3^. The relation between, say, child weight and age is nonlinear, but one could linearize this relation by replacing weight (in KG) by its age-corrected standard deviation score (SDS), or Z-score, and by replacing chronological age with the square root or the log of age. The model is built in the transformed metric. In case predictions in the original scale are needed, one could easily back-transform the result.

The transformation requires the availability of an age-conditional reference standard, that portrays how individuals at a given age vary in their weights. Many different modeling methods exist for constructing such references. See Borghi et al.^4^ for an overview of methodologies. An advisory group for the World Health Organization (WHO) thoroughly reviewed these methods, and recommended application of the Generalized Additive Models for Location Scale and Shape (GAMLSS) by Rigby and Stasinopoulos using the Box-Cox *t*-distribution^4,5^.

The GAMLSS models are flexible regression models where the parameters of the distribution of the response variable are modeled as functions of the independent variables. These models are flexible in the sense that the functions can be linear, non-linear or non-parametric smoothing functions. It models the mean (location), the variance (scale), and the higher-order aspects of the outcomes such as skewness or kurtosis (shape). Location, scale and shape can all change with the predictors. In the Box-Cox *t*-distribution, the response variable has four parameters, the mean (µ), coefficient of variation (σ), the power transformation to symmetry to deal with skewness (ν), and *t*-distribution degrees of freedom to deal with kurtosis (τ). When the latter parameter (i.e. τ) tends to infinity, the Box-Cox *t*-distribution converges to the Box-Cox normal distribution, which is used in the popular LMS method^6^. *LMS* refers to the three GAMLSS parameters that are defined for the Box-Cox normal distribution, i.e. the Lambda for power, Mu for location and Sigma for variance. The model is fitted by maximum likelihood and once the reference model is fixed, one may calculate Z-scores for every weight-age combination.

The observed individual growth curves are often highly irregular due to a variety of causes: early infant growth is an irregular process by itself, the measurement occasions may vary within and between individuals, data errors may result in outliers, there could be measurement errors and so on. On the group level, it makes sense to remove some of this variability by smoothing. When peaks and valleys occur at early measurements we could stretch time by a square root or log transform prior to the smoothing^4^. The values for the parameters of the GAMLSS model determine the degree of smoothing. The appropriate degree of smoothing and the fit of the models can be evaluated using a combination of diagnostic tools described by Van Buuren and Fredriks^7^. The distribution within age (or time) groups can be checked, the Z-score should be normally distributed as N ~ (0,1) at all ages, by testing the Z-scores within age groups and inspecting the detrended quantile–quantile plot (Q-Q plot) of the Z-scores by using worm plots^8^. The shape of the “worms” in the plots indicates the extent to which the data differ from the assumed underlying distribution. Additionally, the shape of the reference curves, i.e. centile plots, can be checked by visual inspections and can be plotted onto the individual data points to detect outliers, gaps in the data and errors in the model. Finding the GAMLSS parameters that achieve the best fitting model is an iterative process for which these diagnostic methods are crucial tools. Especially, the worm plots can be helpful to identify which parameters need to be adjusted to obtain a better fitting model.

### Aligning measurement time-points

In practice, measurements may not be available at all desired time points. In order to be able to model such data, it is vital to align the time points. One way of doing this is to apply linear interpolation or regression^9^. This method assumes that growth has been linear during the interval between the observed data, which may be reasonable if the interval is short, but dubious for longer intervals^10^. A better alternative is to apply the broken-stick model, as pioneered in De Kroon, et al.^11^ and described more fully in Van Buuren^12,13^. The broken-stick model describes a set of individual curves by a linear mixed model using first order linear B-splines. The main use of the model is to align irregularly observed data to a user-specified grid of age breaks. The set of break times are the ages at which the lines connect, so that for each subject we can obtain an estimate at each age break. Because the broken-stick model assumes a linear model, it is best applied to standardized scores relative to a reference standard. The age breaks are chosen by the user as the ages at which the measurements are desired. More information about the assumptions and the model specification can be found in the article on the broken-stick software package^13^.

### Prediction model

The application of the broken-stick model allows us to reorganize the data as a wide matrix, with different time points being represented by different columns. Assuming that the rows of this matrix are independent replications, we may use conventional prediction methods like regression analysis to predict a later outcome given one or more earlier measurements, and potentially adjust for covariates. We may do the modeling entirely in the Z-score scale and transform the predictions afterwards to the original scale, or convert the Z-scores at each age break in the original scale, and develop the prediction model in the original scale. The model can include more or less measurement moments, regulating the amount of information that is known up to a certain time point. Naturally, when the measurement times in the model move closer to the measurement moment that needs to be predicted, the prediction problem becomes easier, provided that the correlation between the measurements increases when time-points are closer. Additional covariates can be used to account for important differences (e.g., between different groups, male/females, and so on), or simply to boost the performance of the predictions. The prediction models thus obtained can be validated by comparing the predicted values to the observed values using the explained variance (R^2^) and the standard deviation of the residuals. Data can be set aside to test the prediction model, and the prediction intervals can be obtained to assess the model performance. The prediction intervals require an adjustment for the broken-stick error when the dependent variable in the prediction model is a broken-stick estimate.

### Ethics approval and consent to participate

Data on the pigs were collected according to Hendrix Genetics protocols, under the supervision of Hendrix Genetics employees. Data were collected as part of routine animal data collection in a commercial breeding program for pigs.

## Application to piglet weights

### Description of data

**Figure 1:**
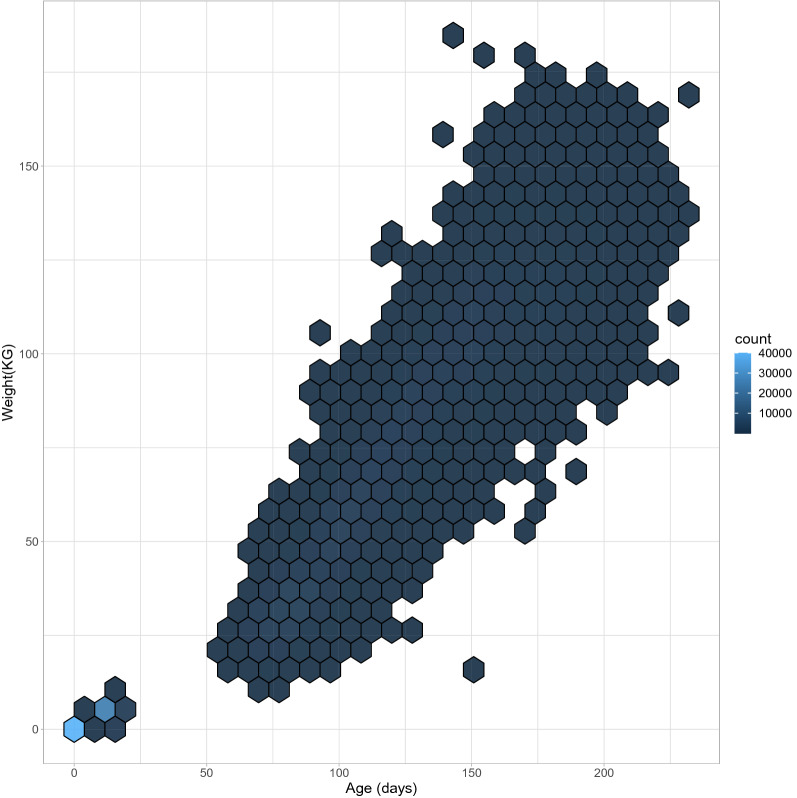
Weight in kilograms for age in days of the piglets measured in 2016.

The application concerns periods of daily measures of piglets weights. The data contains measurements between January 1 2016 and December 31 2016 of 58,917 piglets at 8 different farms. These data were collected as part of routine animal data collection in a commercial breeding program for pigs, according to Hendrix Genetics protocols, under the supervision of Hendrix Genetics employees. The measurement occasions differed between piglets, both in number of occasions and the ages at which the measurements occurred. On average piglets were measured at 4 times with a minimum of 1 measurement and a maximum of 141 measurements per piglet (median = 2). Table 1 presents the descriptive statistics per farm, per breed. Figure 1 displays the piglets weights for all ages and all piglets combined.

**Table 1:** Number of measurements per piglet for each farm and breed.

Farm	Breed	Piglets (n)	Measurements (n)	Measurements per piglet Mean (min–max)
A	1	6940	17,506	2.52 (1–4)
B	1	9697	21,553	2.22 (1–4)
C	2	9496	84,589	9.00 (1–65)
D	1	7732	45,738	6.25 (1–141)
E	1	4531	7426	1.64 (1–2)
F	1	10,615	27,047	2.55 (1–4)
	2	2038	5327	2.61 (1–4)
G	1	273	491	1.80 (1–3)
	2	5314	11,240	2.30 (1–30)
H	2	2281	5294	2.64 (1–41)

### Construction of age-conditional piglet weight references

We fitted a Box-Cox normal distribution (i.e. LMS model) with a mean (µ = 13), coefficient of variation (σ = 8), and a power transformation (ν = 8) to the data. Age was transformed by taking the square root of the age. The model parameters were iteratively determined by inspecting diagnostic tools to evaluate model fit. The fit of the model was explored using worm plots, and visualizing the fitted reference curves and Z-scores. In the modelling phase, a few covariates were considered that could affect the growth pattern: sex, breed, farm of measurement and season of birth. Male and female piglets presented different growth patterns, hence were modelled separately. No differences in the growth pattern were found for the different breeds, and neither for the farms. One of the farms presented a very strong birth season effect, while this was not found for the other farms. Accordingly, the piglets at this farm (i.e. farm C) were not representative for the population and were excluded from being considered in the reference model. .

**Figure 2:**
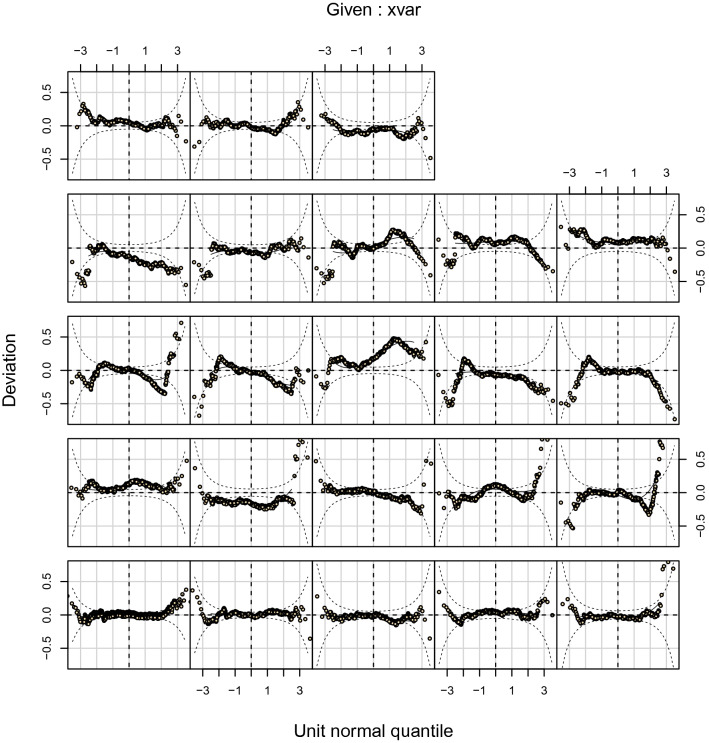
Worm plot for the male piglets for the LMS model. Each panel corresponds to an age bin ordered from bottom-left to top-right.

**Figure 3:**
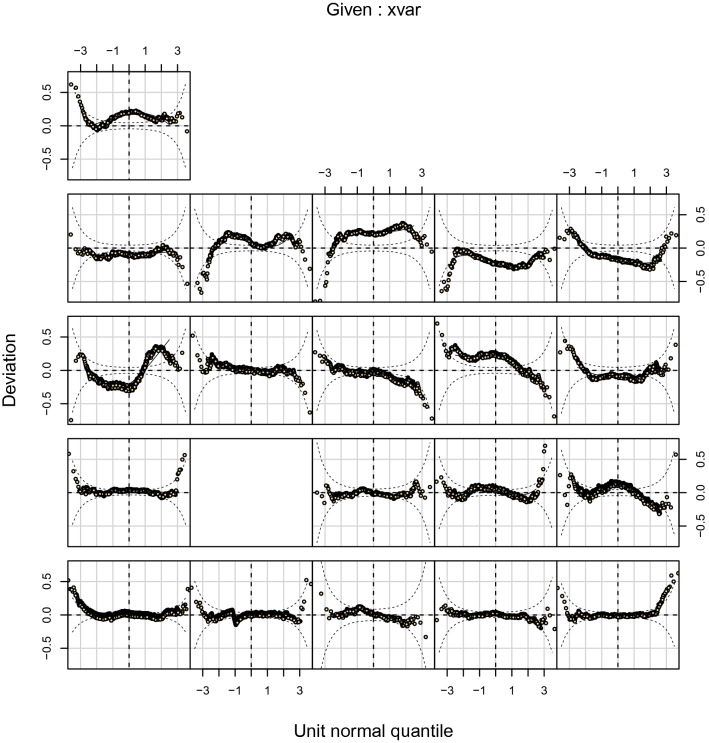
Worm plot for the female piglets for the LMS model. Each panel corresponds to an age bin ordered from bottom-left to top-right, note that the missing panel corresponds to an age bin without observed data.

**Figure 4:**
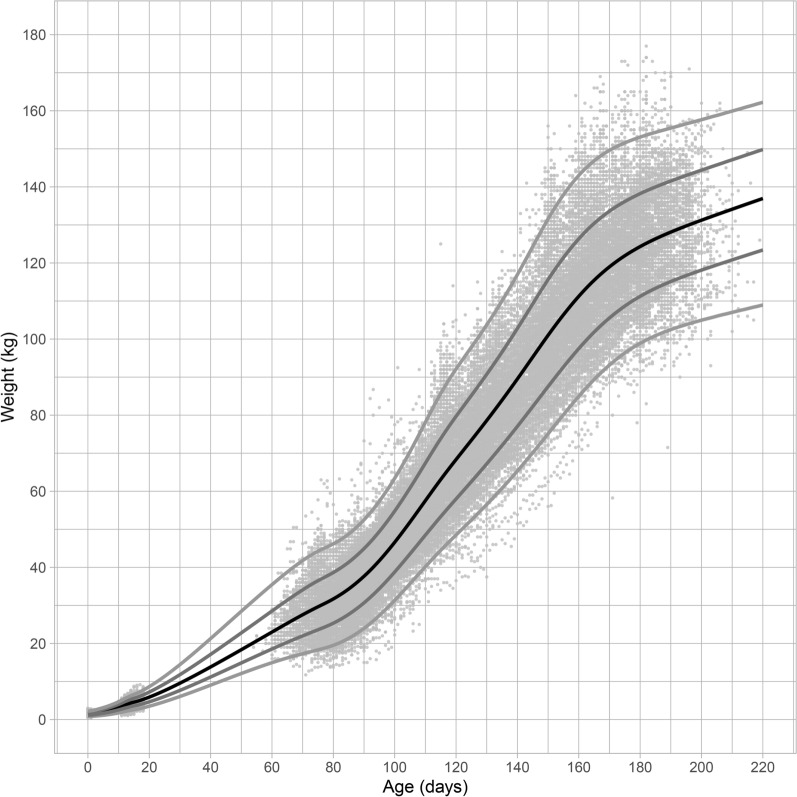
Reference chart for male piglets weight of age in days with the observed data-points. Farm C was excluded. The black line displays the median (50th percentile) the grey lines the 2.3rd, 16th, 84th and 97.7th percentiles, respectively.

**Figure 5:**
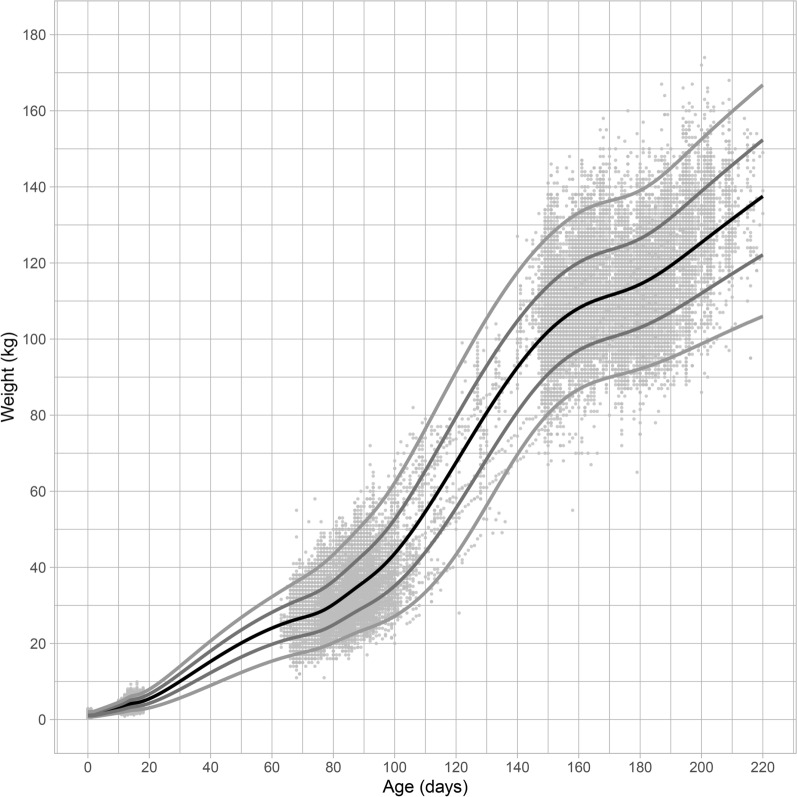
Reference chart for female piglets weight of age in days with the observed data-points. Farm C was excluded. The black line displays the median (50th percentile) the grey lines the 2.3rd, 16th, 84th and 97.7th percentiles, respectively.

**Figure 6:**
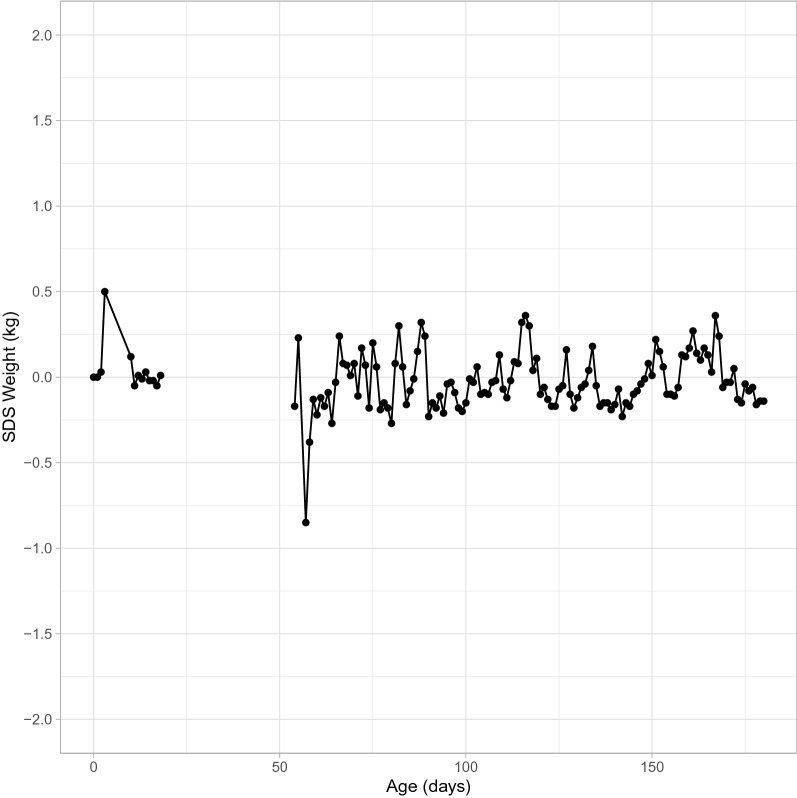
Average Z-scores of weight by age in days for the observed piglet data (excluding farm C)**.**

For the final model, the worm plots (Figs. 2 and 3), and the reference curves plotted on the observed data (Figs. 4 and 5) are presented. The worm plots, shown for 23 age groups, present a good fit of the data to the models for both male and female piglets (Figs. 2 and 3). The data points mostly fit the horizontal axis and show only little deviation. Figures 4 and 5 show that the reference curves follow the observed data nicely. With the reference data, the z-scores can be obtained for the observed data (Fig. 6). The fitted z-scores show that the observed data fit to the model within half a standard deviation. Merely around sixty days the deviation of the observed data to the references from the model is somewhat larger, and between 20 and 50 days there is a gap due to the scarce amount of measurements at these times.

### Aligning measurement time-points

**Figure 7:**
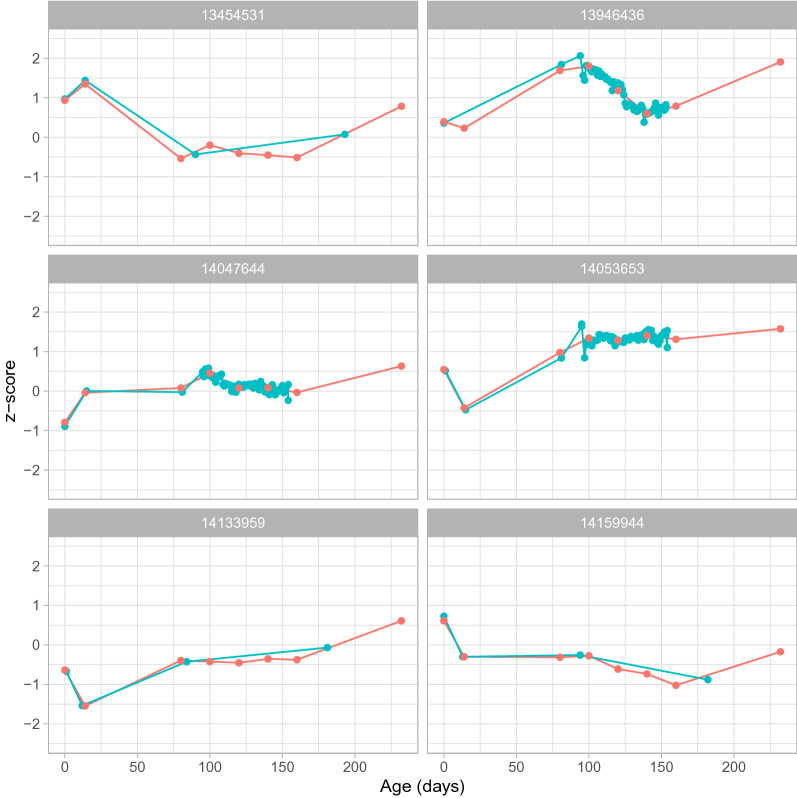
Observed and predicted z-scores for weight resulting from the broken-stick model for six example piglets. The blue dots depict the observed data and the red dots the predicted z-scores from the broken-stick model.

The first step in the broken-stick model is to decide on the age breaks that should be used in the model. For this data, age breaks were set at day 0, 14, 80, 100, 120 140 and 160. This includes two early ages of interest (0 and 14 days), the age at which the prediction should be made (160 days), and ages in-between for which enough data points were available. The broken-stick model was fitted on the Z-scores, using age as the independent variable. Fitting the model in the Z-score scale linearizes the non-linear relation of pig growth with age. The model creates a predicted Z-score for weight at each age break. The assumption of linearity for extreme prediction (e.g. from weight at day 14 to day 160) was checked by inspecting the residuals for nonlinear patterns. Figure 7 plots the observed and modelled weights for six example piglets having very different observed data. The quality of the predictions can be evaluated by the correlation between the predicted z-scores and the observed z-scores, which was 0.997 in our example. The variance of the residuals over all piglets was 0.068, so the broken-stick model represents a very accurate description of the individual growth curves.

### Prediction model

The predicted Z-scores for weight at the break times were back-transformed to the original scale in order to fit prediction models for day 160. Various models were developed for weight at day 160 by extending the age window of the predictors. For each model, weights at previous break times were used as predictors, together with the piglets sex, farm of measurement, breed and season of birth. That way, different predictors are used in each model, depending on the amount of measurement times prior to the dependent weight measurement. For our example data, the break-times are day 0, day 14, day 80, day 100, day 120, day 140 and day 160; so the model for predicting weight at day 160 could contain measurement times at day 0, 14, 80, 100, 120 and day 140 or only day 0 and 14 for example as Eq. (1):

1 $${W}_{160}={W}_{\{t|t<160\}}+Breed+Farm+Sex+Season$$

where ${W}_{160}$ represents the weight at day 160; ${W}_{\{t|t<160\}}$ are the weights at the break-times before the 160th day.

In addition, models were developed to predict the weight gain at various intervals, so the weight difference between two time points was also used as outcome. Again, we used the preceding time-points as predictors for these models, as well as the piglets sex, farm of measurement, breed and season of birth.

2 $${\Delta W}_{y2,y1}={W}_{\{t|t\le y1\}}+Breed+Farm+Sex+Season$$

where ${\Delta W}_{y2,y1}$ represents the weight difference between two adjacent age breaks; ${W}_{\{t|t\le y1\}}$ are the weights at the preceding age break. For example: ${\Delta W}_{\mathrm{160,140}}$ is the weight difference between day 160 and day 140, which can be predicted by the break-times at day 0, 14, 80, 100, 120 and 140 (and the covariates).

In Table 2, the model performance for predicting weight at day 160 are presented for a decreasing amount of break-times as predictors. The R^2^ decreases when the predicting age breaks are further away from the outcome day 160. Moreover, the standard deviation of the residuals increases. Nevertheless, even with only information up to day 80, still more than 77% of the variance at day 160 is explained. A similar trend can be observed in Table 3, where the model performance for predicting the weight differences are presented.

**Table 2:** Results of prediction models for weight at day 160 with decreasing amount of measurement history used. Covariates: breed code, farm of measurement, sex, season of birth.

Measurement history (days)	Explained variance (R^2^)	Standard deviation of residuals (in KG)
≤ day 140	0.990	1.044
≤ day 120	0.960	2.135
≤ day 100	0.861	3.982
≤ day 80	0.773	5.098
≤ day 14	0.496	7.593
Day 0	0.494	7.611

**Table 3:** Results of prediction models for weight gain with decreasing amount of measurement history used. Covariates: breed code, farm of measurement, sex, season of birth.

Measurement history (days)	Weight gain period (days)	Explained variance (R^2^)	Standard deviation of residuals (in KG)
≤ day 80	80–100	0.766	1.555
≤ day 14	80–100	0.581	2.082
day 0	80–100	0.489	2.299
≤ day 100	100–120	0.555	1.549
≤ day 80	100–120	0.280	1.971
≤ day 14	100–120	0.196	2.083
day 0	100–120	0.191	2.089
≤ day 120	120–140	0.817	0.907
≤ day 100	120–140	0.639	1.274
≤ day 80	120–140	0.638	1.277
≤ day 14	120–140	0.635	1.281
day 0	120–140	0.607	1.330
≤ day 140	140–160	0.911	1.044
≤ day 120	140–160	0.837	1.415
≤ day 100	140–160	0.869	1.686
≤ day 80	140–160	0.760	1.718
≤ day 14	140–160	0.756	1.731
Day 0	140–160	0.747	1.765

### Validation with new data

Furthermore, the model performance was tested on data from 241 piglets that were born in October 2016 and have data for at least 160 days. This selection includes measurements for ages that go beyond the age range that was used in the modelling data. For these test piglets we transformed the weights to Z-scores using the model references and subsequently fit the broken-stick model to align the measurements. In the next step we run the prediction model to predict weight at day 160 from the observations at day 0 and day 14 only. For the validation we indicated whether the observed weight at day 160 was within the prediction interval for the predicted weight at day 160. The prediction model uses the broken-stick estimate for day 160 as the dependent variable. In that case, the prediction intervals require an adjustment for broken-stick error. Usually, prediction intervals are computed by using the standard deviation of the model residuals. As presented in Table 2, in our model this is equal to 7.593 KG, or reported as residual variance 0.516 (i.e. 1−R^2^). In our case we need to add the error variance due to misfit of the broken-stick model defined as the residual variance of the broken-stick model, i.e. 0.068. The total residual variance becomes 0.516 + 0.068 = 0.574. This variance corresponds to a standard deviation in KG at 160 days equal to 122.6 KG (0.575 SD)−111.2 KG (0 SD) = 11.4 KG. Accordingly, the prediction intervals are defined in Eq. (3).

3 $${W}_{160}\pm {t}_{(1-\frac{\alpha }{2},n-2)}x\,{SE}_{prediction}$$

where ${W}_{160}$ is the predicted weight for an animal, the t-multiplier for the 50% prediction interval is 0.674 and the ${SE}_{prediction}$ is 11.383 KG.

**Figure 8:**
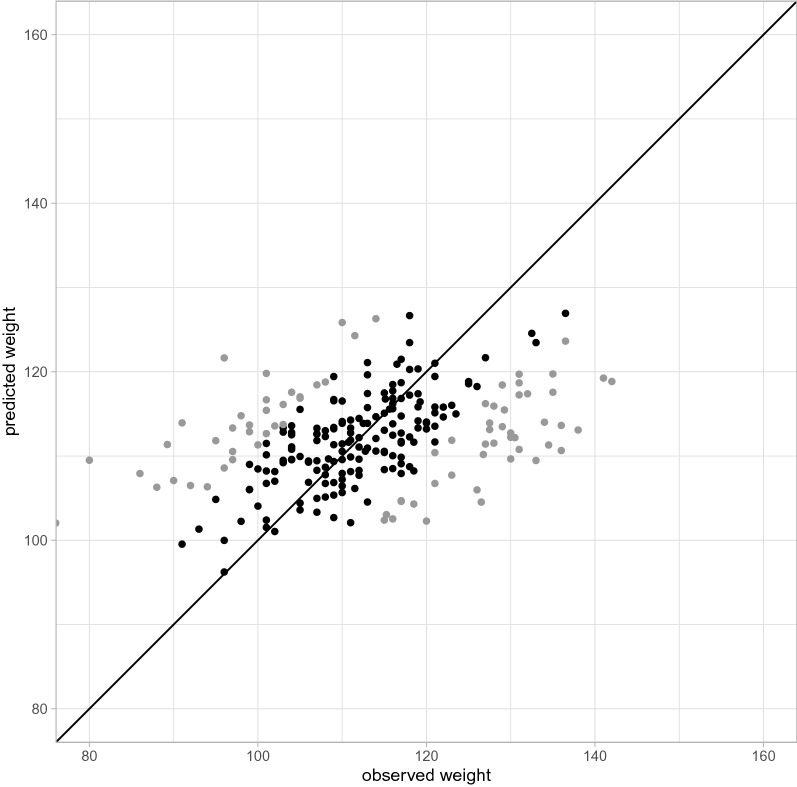
Observed weights against the predicted weight at day 160 from observations at day 0 and day 14 for 241 test piglets. Black dots indicate the observed weights within the 50% prediction interval of the predicted weights.

We found that for 53% of the test-piglets, the observed weight at day 160 was within the 50% prediction interval. In Fig. 8 the observed weights are plotted against the predicted weights. The solid diagonal line displays a perfect prediction of the observed weight at day 160.

## Discussion

In this paper we outline a simple three-step process for developing a prediction tool for (daily) monitoring data, and illustrated the application of these steps to piglets weight data. In step 1 references were developed to account for nonlinearities in the data. Step 2 aligns the measurement occasions across subjects, and step 3 constructs and evaluates prediction models by applying conventional regression techniques to the wide data. After each step, performance of the modelling step can be monitored using several visualization tools and statistics.

In the first step, creating the references, it is important to consider a representative sample for the reference standard that is to be developed. In our illustration data, one of the farms operated under different (not-representative) conditions compared with the usual piglet farms. For that reason, it was decided to exclude that farm for creating the references. In the next steps, however, this farm was included again, so that the data could be used in the prediction model. Additionally, fitting the model to estimate the reference standard should be done with care, making sure that the data fits the model well at the entire age range. As shown in the example, worm plots can help to diagnose model fit at separate age blocks. This process may require fitting several different models in order to find the model with the best fit to the data. Furthermore, as mentioned previously, the selection of the break-times is one of the most important parts of the broken-stick model. This requires consideration of both substantive interest (i.e. what time points are of interest for the prediction model) and the availability of data. The diagnostics of the broken-stick model to the data can be utilized to make sure that the break-times and model fit the data well. Additionally, also in the final step it is important to evaluate the models and evaluate whether the explained variances are sufficient for the models to be used in the final tool.

In this paper, the methodology was illustrated by an application to data collected in animal breeding. Nevertheless, the presented methodology can be applied in many other fields. Actually in any field where repeated measurements are performed, the methodology can be applied to develop a tool to obtain predictions from previous measurements. Additionally, after the first two steps, instead of prediction models, important associations or growth patterns can be evaluated, to steer dynamic processes over time. Accordingly, the methodology described has many potential uses and can be modified to fit the substantive interest.

### Conclusions

As discussed, it is very important to apply each of the three steps carefully and use the diagnostic tools that are available for each step. The described methodology can be composed into a tool that deals with intensive irregular longitudinal data, while the available information is used in an optimal way. With the resulting tool, future outcomes can be accurately predicted, as shown for the example of piglet weights. The methodology is adaptable to many different applications.

## Data Availability

The data used in this study have been collected as part of the routine data collection within the swine genetic program of Hendrix Genetics. These data contain individual measurements of piglets body weight over time. The data have been used to assess genetic value of individual animals for the purpose of selecting the next generation of elite animals. The supple of genetically improved swine is a highly competitive business, and this data could potentially reveal some trade secrets to competitors. For that reason, Hendrix Genetics does not wish to make this data publicly available. The data can be made available to interested researchers after consultation with Abe Huisman (P.O. Box 30, 5830 AA Boxmeer, The Netherlands; T:+31485801951), director of R&D Business Unit Swine of Hendrix Genetics.

## Abbreviations

GAMLSS: Generalized Additive models for location scale and shape
KG: Kilo grams
LMS: Lambda, Mu, Sigma parameters that summarize the measurement’s distrubution
Q-Q plot: Quantile–Quantile plot
SDS: Standard deviation score
WHO: World Health Organization
